# COSMAS: a lightweight toolbox for cardiac optical mapping analysis

**DOI:** 10.1038/s41598-021-87402-9

**Published:** 2021-04-28

**Authors:** Jakub Tomek, Zhinuo Jenny Wang, Rebecca-Ann Beatrice Burton, Neil Herring, Gil Bub

**Affiliations:** 1 Department of Physiology, Anatomy, and Genetics, University of Oxford, Oxford, UK; 2 Department of Computer Science, University of Oxford, Oxford, UK; 3 Department of Pharmacology, University of Oxford, Oxford, UK; 4 Department of Physiology, McGill University, Montréal, Canada

**Keywords:** Data processing, Image processing, Software

## Abstract

Optical mapping is widely used in experimental cardiology, as it allows visualization of cardiac membrane potential and calcium transients. However, optical mapping measurements from a single heart or cell culture can produce several gigabytes of data, warranting automated computer analysis. Here we present COSMAS, a software toolkit for automated analysis of optical mapping recordings in cardiac preparations. COSMAS generates activation and conduction velocity maps, as well as visualizations of action potential and calcium transient duration, S1-S2 protocol analysis, and alternans mapping. The software is built around our recent ‘comb’ algorithm for segmentation of action potentials and calcium transients, offering excellent performance and high resistance to noise. A core feature of our software is that it is based on scripting as opposed to relying on a graphical user interface for user input. The central role of scripts in the analysis pipeline enables batch processing and promotes reproducibility and transparency in the interpretation of large cardiac data sets. Finally, the code is designed to be easily extended, allowing researchers to add functionality if needed. COSMAS is provided in two languages, Matlab and Python, and is distributed with a user guide and sample scripts, so that accessibility to researchers is maximized.

## Introduction

Optical mapping of explanted hearts or cell cultures is an integral part of experimental cardiac research, allowing detailed observation of action potentials (AP) and calcium transients (CaT) in cardiac preparations^1–3^. It contributed to our understanding of arrhythmogenesis with regards to, e.g. reentrant spiral activation^4^, autonomic modulation^5,6^, post-infarction conduction abnormalities^7^, cardiac alternans^8,9^, and reentry following acute myocardial infarction^10^. While optical mapping offers excellent spatial resolution of the recordings compared to electrophysiological techniques such as monophasic AP mapping or electrode arrays, the hundreds to thousands of signal traces produced in a single optical mapping recording generally require automated computer-based processing.

Despite the widespread use of optical mapping techniques in cardiac research, there are surprisingly few standardized tools for their analysis. Both available software packages, Rhythm^11^ and the recently published ElectroMap^12,13^ are relatively complex, generally require a large amount of parameters to be set, and are controlled via sophisticated graphical user interface (GUI). A major advantage of GUI-controlled software is that even a person with no programming or data analysis background can use it to analyze data. On the other hand, GUI based software greatly limits its use in a wide variety of scenarios, such as headless operation—i.e. running remotely on a server, cluster or cloud without a GUI. Indeed, the need for flexible batch processing was one of the motivations for rewriting ImageJ^14^. In the specific context of cardiac data analysis, the GUI approach presents multiple limitations. First, it exposes the user to a wide range of functionality, boxes for setting of a wide array of parameters, and method selectors, many of which may be irrelevant for a particular task the user tries to do. This may be overwhelming particularly for the users with low data analysis experience, for whom it may be difficult to identify and select appropriate methods and parameters. Second, the software architecture of GUI tools is often dictated by the GUI handling code, which makes it hard to extend or modify them when the pre-built functionality is insufficient. Ultimately, Rhythm and ElectroMap require separate processing for each recording analyzed, not supporting batch mode processing. This can work well for analyzing a small number of files (especially with ElectroMap’s helpful option to store and load a set of processing parameters) but can quickly become a bottleneck for a larger-scale analysis. Optical mapping can easily produce hundreds of recordings in a single sequence of experiments, and manually loading, processing, and storing the results using a GUI is rather time-consuming. This is particularly true when one realizes, midway in the analysis, that the processing parameters need to be changed, and the whole process needs to be started over.

In this article, we present a new software toolkit COSMAS (COmb-based Software for optical-Mapping AnalysiS) for analysis of cardiac optical mapping data, such as from Langendorff hearts or in vitro cardiac cell cultures. It is built around the comb algorithm for detection of APs or CaTs in recordings with known activation rate (e.g. under external pacing or regular sinus rhythm)^15^, offering a high quality of processing in a range of common tasks arising in optical mapping of membrane potential or calcium. This includes activation mapping, conduction velocity extraction, mapping of AP and CaT duration, S1-S2 protocols, and processing of duration and amplitude alternans. COSMAS is designed to be used via straightforward scripting, avoiding the complexity of GUI, being simple (633 lines of code versus 9115 of ElectroMap, excluding comments, empty lines, and external libraries), and naturally supporting batch processing.

Two functionally identical versions of COSMAS are provided, one implemented in MATLAB, and one in Python 3, giving the user choice of  which language to use. This is in contrast with Rhythm and ElectroMap, which both require MATLAB (ElectroMap can be run as a stand-alone, but it cannot be adjusted in this form).

## Methods

**Figure 1:**
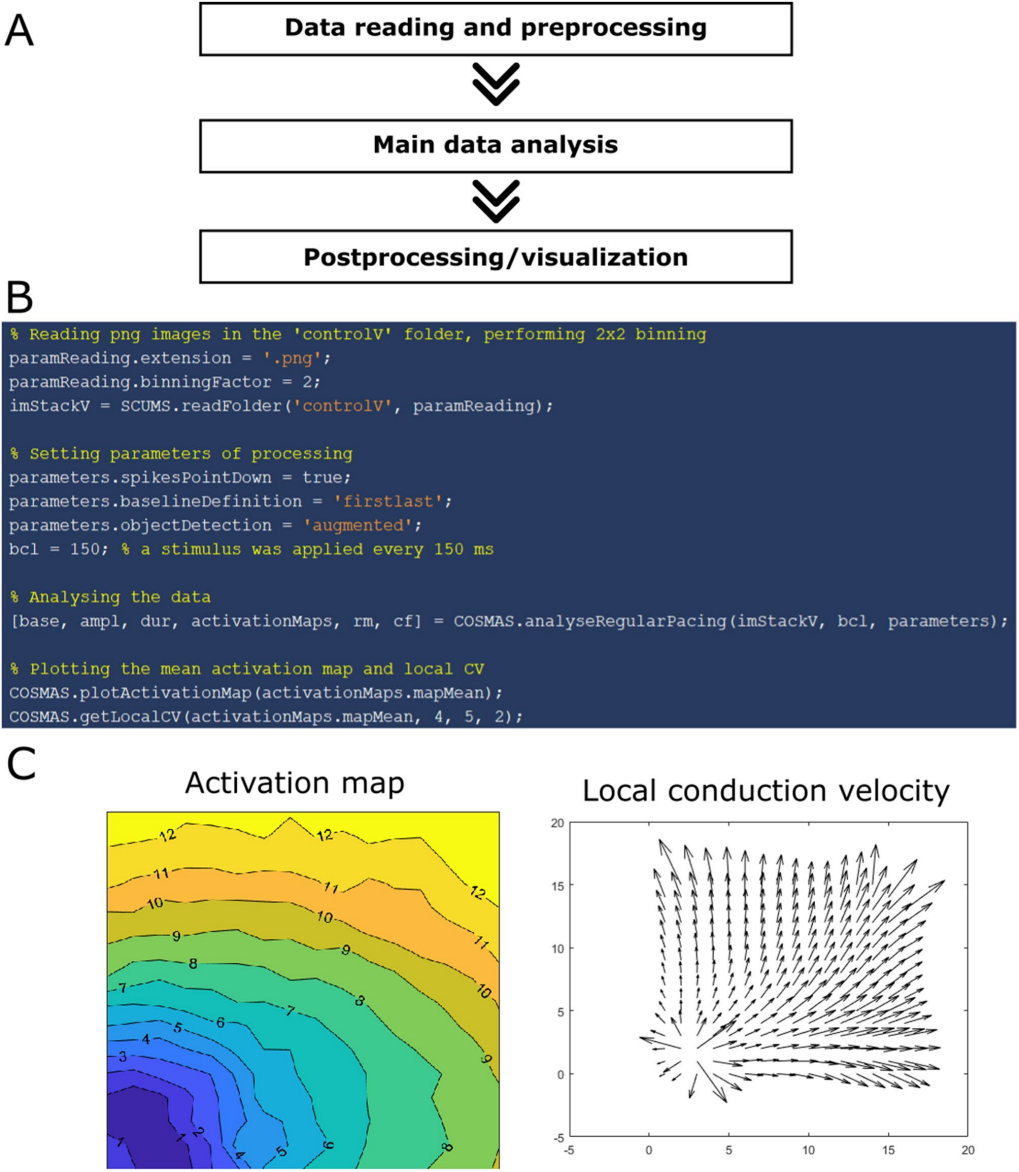
Illustration of COSMAS use. (**A**) General structure of a processing script, (**B**) a concrete example of a script in Matlab, (**C**) the figures produced, showing isochronal activation lines (in ms) and local estimation of conduction velocity.

The general structure of analysis using COSMAS is given in Fig. 1A. A concrete sample script computing and plotting a map of average activation pattern is shown in Fig. 1B, with the figure produced in Fig. 1C. A common practice in our lab is to run similar code in a for-loop over all recordings from a sequence of experiments, extracting the features of interest, and then having the results conveniently stored in Matlab variables, ready for further processing and/or visualization. Below is described the functionality provided by COSMAS for the steps involved in processing optical mapping data.

**Figure 2:**
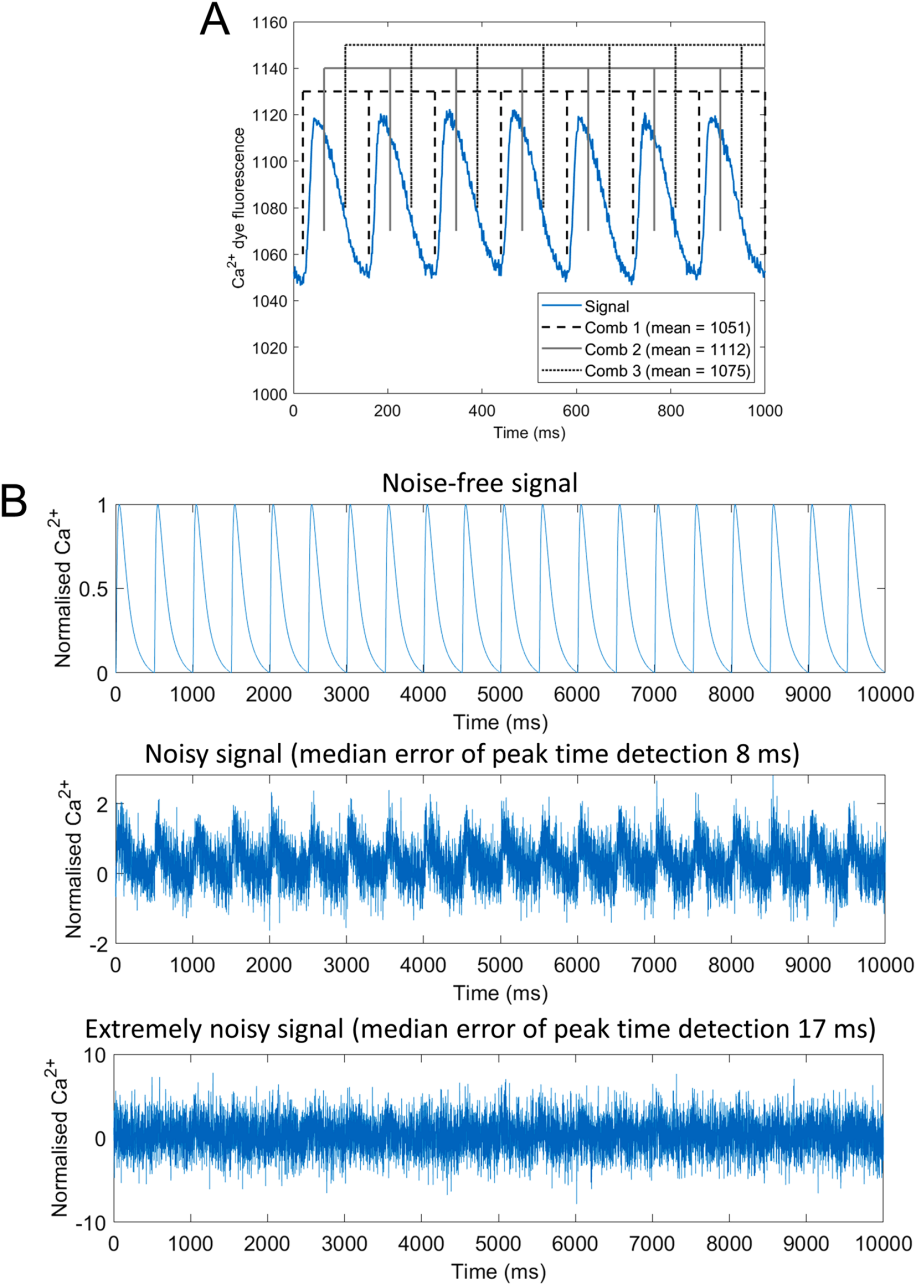
The comb algorithm and its noise-resistance. (**A**) Illustration of comb positioning in a recording of calcium transients in rat heart paced every 140 ms. Three distinct positions of comb are shown, with the average value under comb teeth being given in the legend. The dashed comb has the lowest average value, being the best comb position for extraction of signal minima. (**B**) An example of the trace of 20 calcium transients (generated using the computer model ToR-ORd^16^) with no added noise (top), heavy noise (center), and an extremely high amount of noise added (bottom). COSMAS detected calcium transient peak times with a median error of only 8 ms for heavily noisy signal and only 17 ms in the extremely noisy signal, demonstrating the robustness of the algorithm to noise. Further quantitative data on noise-resistance versus the number of beats in a recording are given in Supplementary Figure 1.

COSMAS makes extensive use of the comb algorithm^15^, which is a local-minima-finding algorithm that leverages the knowledge of the activation pattern of the recording (the algorithm description is included in Supplementary Materials). The high-level intuition of the algorithm, illustrated in Fig. 2A, is that a ‘comb’ is constructed with teeth at predefined distances apart (e.g. if pacing stimuli are applied 150 frames apart, the comb has teeth 150 frames apart as well), and it is slid over the recording, measuring the average signal under the teeth. Subsequently, the position of comb with the lowest average signal is selected, and a small-radius local search is carried out around each comb tooth (within ‘refinementWidth’ ms around the initial location), selecting the ultimate signal minima locations. This approach avoids a range of artefacts inherent to threshold-based approaches and is highly resistant to noise (Fig. 2B, Supplementary Figure 1). In addition, the comb-detected minima are guaranteed to be spaced correctly with regards to the known activation rate. Approaches not using this (such as variants of thresholding) can spuriously detect action potentials too close or too far from each other.

### Data reading and preprocessing

COSMAS has two functions for reading data, reflecting the two most common imaging data formats: TIF stack (with slices corresponding to single frames), and an image sequence in a folder (TIF, PNG, or JPG images can be read). Both functions return a 3D matrix representing the recording, with the third dimension corresponding to time coordinates. The user can use alternative means of loading nonstandard or proprietary formats, as long as the result is in the same form of 3D matrix. This general approach makes COSMAS readily useful for any kind of optical mapping data.

In addition to reading the data, the user has the option to carry out spatial binning on each frame of the data read, trading reduced spatial resolution for improved signal-to-noise ratio.

Subsequently, a binary mask may be applied to each frame of the stack using COSMAS, which is useful to discard irrelevant pixels such as the area around a heart, for Langendorff heart imaging, or the area around a dish with a cell culture. COSMAS also contains a function to provide a maximum-contrast averaged image of the recording, which may be useful for detecting such areas and drawing the mask.

Given the easy-to-interpret representation of a recording, the user may apply any additional filtering not implemented in COSMAS that they implement on their own.

### Main analysis

The analysis of a recording is carried out slightly differently for cases when the recording contains multiple passes of a wave (at least three) than when it contains only a single wave pass. In both cases, the user is provided with spatial and temporal information on activation pattern (and thus conduction velocity), signal amplitude, duration, or baseline, as well as a map of signal recovery (e.g. repolarization for AP data). There is also a third ‘hybrid’ option, combining both approaches.

### Multi-wave processing

**Figure 3:**
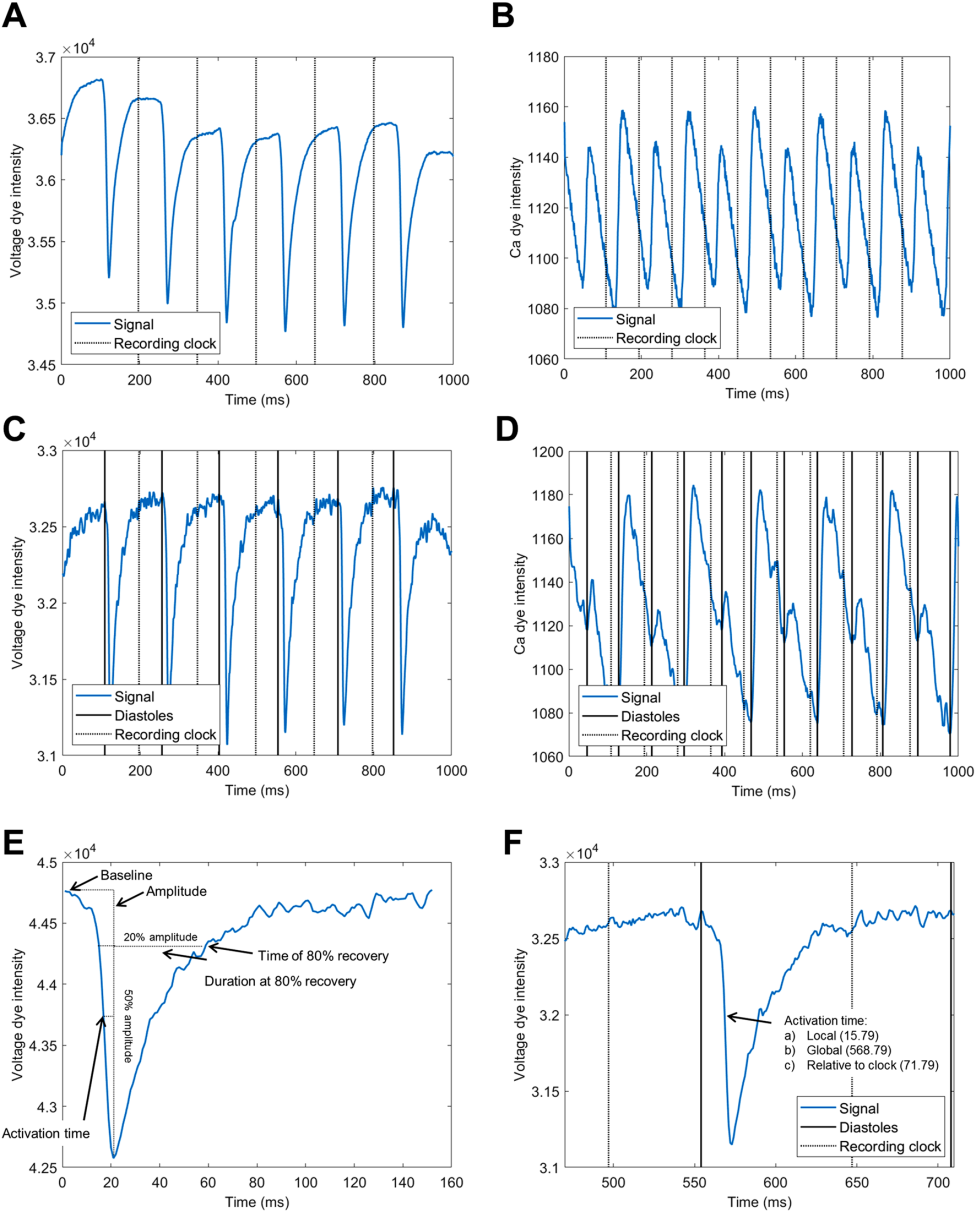
Illustration of stimulus segmentation and feature extraction. (**A**) Average trace of a voltage mapping recording in rat heart at 130 ms basic cycle length (the lower the intensity, the higher the membrane potential), showing the recording clock (half-points between peak activations). (**B**) Average trace of a calcium mapping recording in rat heart at 85 ms basic cycle length (the higher the intensity, the higher the calcium concentration), showing amplitude alternans. (**C**) Single-pixel trace from the recording used in (**A**), also showing the points corresponding to the points of smallest activation (“diastoles”, solid black lines), as well as the global clock (dotted black lines). (**D**) Single-pixel trace from the recording in (**B**), showing pronounced calcium alternans. This recording illustrates, among other things, that despite baseline subtraction and signal smoothing, no single threshold can be used to satisfactorily segment distinct CaTs, while the comb-based approach of COSMAS correctly identifies CaTs as the intervals between diastoles. (**E**) Illustrations of features extracted on a single rat AP. (**F**) Illustration of how activation times are treated. Within single activation, the local activation time is first extracted (15.79 ms after the preceding diastole). This corresponds to the global time (within the whole recording) of 568.79 ms, from which the time of the preceding clock boundary is subtracted, and COSMAS records that the activation came 71.79 ms after the preceding clock. In this way, even though each pixel’s trace may have diastoles detected at different times, all pixels are synchronized to the same global clock, which allows extraction of meaningful activation maps for each pass of a cardiac wave.

The general approach is that individual wave passes are segmented in the recording and the properties of each wave pass are measured separately. They are then provided to the user in both the raw form and as an averaged value over the entire recording. The concrete steps are as follows:

1. A “recording clock” is obtained on the spatial average trace of the whole recording as the set of midpoints between peak activations (Fig. 3A,B, dotted lines). Signal peaks in the averaged curve are found using the comb algorithm. COSMAS contains a switch allowing the extraction of peak activations either as signal maxima (for calcium imaging) or as signal minima (arising in voltage mapping). The recording clock is not meant to provide a good segmentation of single APs and CaTs, but merely to provide a global set of “bins” to which recorded data are later assigned in a consistent way. In this way, COSMAS keeps track of the specific wave pass to which an extracted feature belongs.
2. For each pixel in the recording, its trace over time is extracted from the 3D matrix representing the recording. The following processing and analysis functions is then applied on this trace:
  - It may be smoothed using the Savitzky-Golay filter, which was chosen for its capability of removing noise while preserving genuine sharp changes in signal intensity, such as AP upstrokes.
  - Temporal baseline drift may be subtracted using polynomial smoothing. If this is carried out, the mean of the original signal is re-added after the baseline subtraction. In this way, the general offset of the recording is maintained, allowing e.g. the monitoring of photobleaching as the reduction in the signal baseline over consecutive recordings.
  c. Signal diastoles (points of least activation) are extracted via the comb algorithm, which supports both upward- and downward-pointing signal, and the diastoles separate the signal into single cardiac activations (APs/CaTs) (Fig. 3C,D). While the comb algorithm by default supports only regularly activated recording, the user may also provide a custom comb with arbitrary distances between ‘comb teeth’, which can be used to process even irregularly activated recordings. For every single cardiac activation found using the comb, the following features are extracted (illustrated in Fig. 3E):
    i. Signal baseline is extracted as the first value or the average between the first and the last values, as determined by the user.
    ii. The amplitude of the activation is computed as the difference between peak of the signal and the baseline.
    iii. Cardiac activation duration at the user-defined level of recovery is obtained via thresholding (providing, e.g., AP duration at a given level of repolarization). Linear interpolation is used at the threshold crossing with the signal to obtain a more precise value. The recovery time at the given level is also stored (corresponding, e.g., to the time at which 80% recovery was achieved in an AP), allowing the study of dispersion of repolarization.
      It may occur that the signal segment between two points of a recording clock crosses a given threshold at multiple points in time, leading to multiple objects being segmented, even though there is only one which is true cardiac activation. This usually arises from large amount of noise, or from motion artefacts. COSMAS offers three options for selecting the segmented object from which the features are extracted:
    1. ‘first’—the first segmented object in the segment is taken
    2. ‘largest’—the segmented object with the longest duration is taken
    3. ‘augmented’—in this case, the information on the time of maximum activation rate in each AP/CaT is used to augment the decision making about which segmented object to select:
      - The derivative of the whole smoothed trace (i.e. before it is split into segments corresponding to single activations) is approximated as the difference between consecutive values.
      - Subsequently, the comb algorithm is applied to this signal to find the times of peak activation rate (corresponding to AP or CaT upstrokes). Thanks to the comb algorithm working on the whole trace, this is much more robust to noise than selecting the peak activation rate in a single AP/CaT only.
      - When the ‘augmented’ object detection is then used for a trace segment corresponding to a single activation, the time point of the peak activation rate in this segment (coming from the previous step) is extracted (there is exactly one) and the segmented object with smallest distance from this point is selected.
  iv. Activation time (used for activation mapping) is estimated as the time when half-maximal amplitude of the cardiac activation is achieved (again using linear interpolation to obtain a precise estimate).
  v. The activation time is subsequently converted to global time coordinates (i.e. not just within the single cardiac activation, but within the whole recording), and the nearest global recording clock time that precedes the activation time is subtracted from it (Fig. 3F). In this way, the activation times of all single pixels are synchronized using the global recording clock, allowing the extraction of meaningful activation maps. The time of recovery of cardiac activations extracted in the previous step is normalized to the global clock in the same way.
  vi. The extracted features (baseline, amplitude, duration, recovery time, and activation time) are assigned to an appropriate “bin” that is bracketed by two recording clock markers. This is done by taking the half-time of the single activation and seeing in which bin in the global recording clock it belongs. The activation is ignored if its half-time falls before the first clock boundary, or after the last one, to prevent processing of potentially incomplete activations at the start/end of the recording.
3. Spatial maps of all the features are returned to the user, along with their spatial and temporal (over beats) averages.

#### Single-wave processing

COSMAS also contains functionality to process recordings where only a single pass of a wave is recorded. The processing of such data is analogical to the step 2c of the multiple-wave processing. No separation into distinct waves is carried out, and all the features are extracted on the whole duration of the recording (i.e. there is no assumption on the rate of activation in this case). Given that a single wave is present, no averaging over multiple passes is performed.

#### Hybrid processing

A third option for processing the data is to split a regularly-activated multi-wave stack into smaller substacks, with each corresponding to a single pass of the cardiac wave, which are subsequently averaged and processed using the functionality for single-wave processing (Supplementary Figure 2). The first point of splitting is extracted as the first point of the recording clock (as in multi-wave processing, step 1), with further splitting points being placed at multiples of basic cycle length after that. In this way, all sub-stacks contain a length of signal corresponding to a single basic cycle length, and the signals are naturally aligned, allowing direct averaging without the need for further alignment.

The hybrid processing is advantageous for signal with severe noise perturbation, occasional movement artefact or instability, which could give artefactual outputs for one or several wave passes in the multi-wave processing approach. At the same time, this approach loses the temporal information of multiple wave passes which can be analyzed separately and it does not allow analysis of recordings where signal features change substantially over time (e.g. alternans).

### Postprocessing

COSMAS returns all of its outputs in a form that is easy to interpret straight away, with matrices representing spatial maps of features and averaged values of these, but it also has several additional functions facilitating the most common post-processing tasks. First, a function to plot activation maps is provided (utilizing the function ‘contourf’ in Matlab and ‘matplotlib.pyplot.contour’ in Python, both with default parametrization). Second, a function for estimation of conducting velocity (CV) is provided, allowing the measurement of CV between multiple points in an activation map. Third, a function to compute local CV and the resulting conduction vector field as per the method by Bayly et al.^17^ is provided. Fourth, it contains a function to generate maps of alternans in any mapped feature, such as duration, amplitude, etc. When alternans is processed, the feature maps corresponding to even passes of a wave through the heart/dish are averaged, and the same is done for odd wave pass maps. Based on these even/odd maps, alternans behavior in each pixel is defined as either ‘large-to-small’ or ‘sMAPE’ (symmetric mean absolute percentage error), which are defined below.

The ‘large-to-small’ alternans is defined, for each pixel (i,j), as $\frac{\mathrm{max}(ma{p}_{even}\left(i,j\right), ma{p}_{odd}\left(i,j\right))}{\mathrm{min}(ma{p}_{even}\left(i,j\right), ma{p}_{odd}\left(i,j\right))}$., i.e. the ratio of larger-to-smaller feature (e.g. CaT amplitude) value between even and odd wave passes.

The ‘sMAPE’ alternans is defined, for a pixel (i,j), as:

$$\frac{|ma{p}_{even}\left(i,j\right)- ma{p}_{odd}\left(i,j\right)|}{ma{p}_{even}\left(i,j\right)+ ma{p}_{odd}\left(i,j\right)}$$

### Computational performance

Using a new PC with AMD Threadripper 3970X and a fast SSD, the Matlab version of COSMAS can process a single 1000-frame recording of 32 × 32 pixel images (binned to 16 × 16) in under a second, including loading the data and carrying out all analyses (the runtime is ca. 2 s in the Python version of COSMAS). On an older computer with Xeon 1650v3 and magnetic drive (7200 rpm), the runtime per such recording is ca. 2.4 s in Matlab. The analysis code is encapsulated in a function and it is straightforward to analyze multiple recordings in parallel, further reducing the runtime.

### Comparison with ElectroMap

When making a comparison of outputs between COSMAS and ElectroMap^12^, simulation parameters were made as similar as possible. When generating data for Fig. 9 in “Results” section, both tools used fourth order polynomial baseline subtraction, Savitzky-Golay filtering with window size of 11, and no spatial filtering. The ‘hybrid’ activation detection was used in COSMAS, which best corresponded to ElectroMap’s activation detection method where multiple wave propagations were averaged before the detection process.

When generating data for Fig. 10 in “Results” section, parameters of signal pre-processing were again fourth order polynomial baseline subtraction, Savitzky-Golay filter with window size of 11, and no spatial filtering. Multi-wave processing type was used in COSMAS (being the only approach suitable for alternans analysis), and a corresponding method of analysis was used in ElectroMap, avoiding averaging of multiple CaTs before their processing. The CaT “release” alternans was computed in ElectroMap, which corresponds to what COSMAS reports; baselines in both COSMAS and ElectroMap were taken as the first value in each segmented CaT. When computing alternans magnitude, ElectroMap uses the following formula: $1-\frac{Ca amplitud{e}_{smaller}}{Ca amplitud{e}_{larger}}$, and the same was computed using COSMAS for the purpose of comparison in Fig. 10.

When testing the effect of noise on processing in both tools, the raw data were perturbed with Gaussian noise of a given standard deviation (the same data were then used for both tools).

### Data description

**Figure 4:**
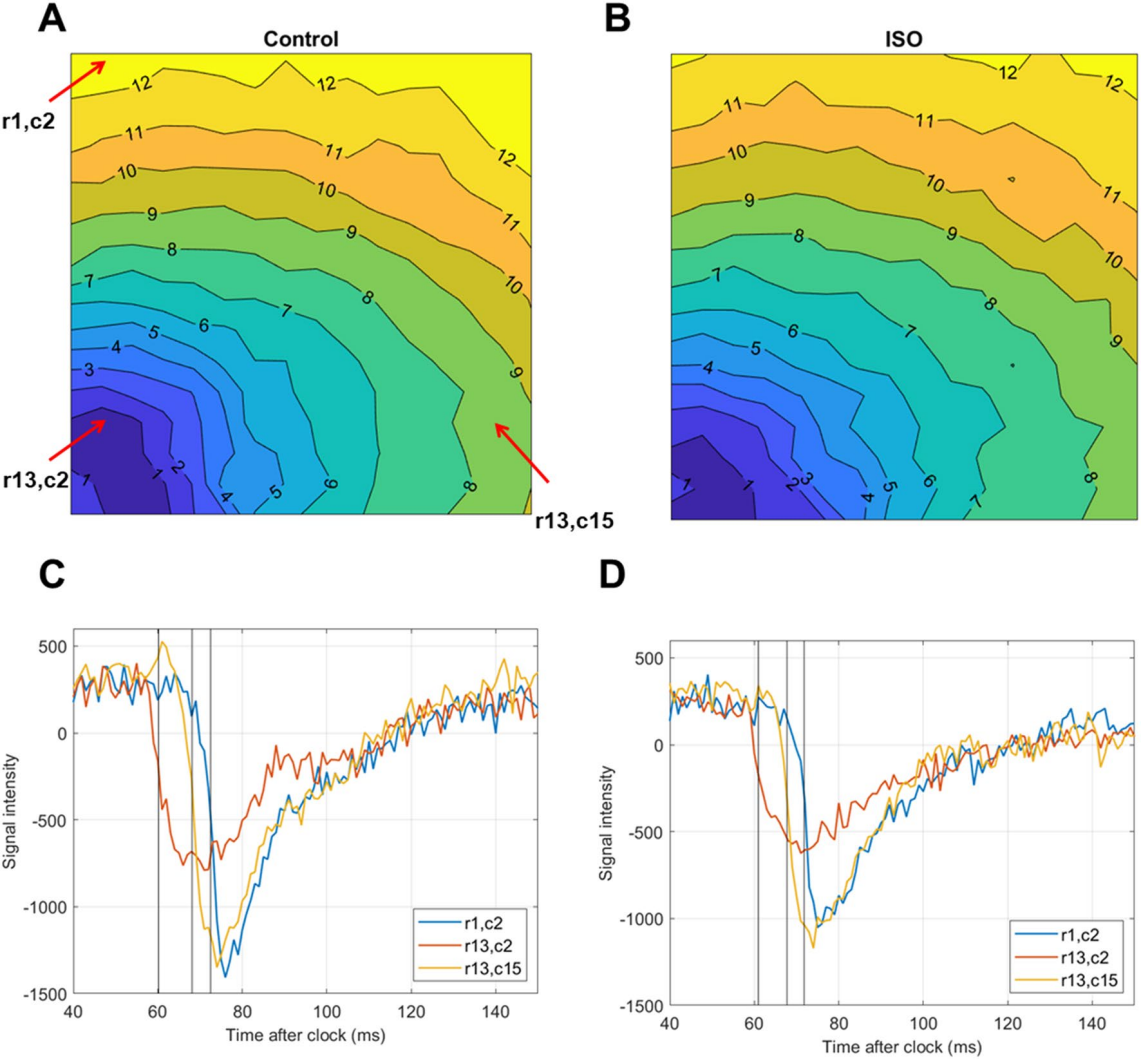
Activation mapping in a voltage-mapped Langendorff rat heart before and after isoproterenol application. (**A**) Activation map from a wave pass in control condition; isochronal lines are given in milliseconds, and the map is visualised with a minimum activation time of 0. Spatial resolution is 16 × 16 pixels. Red arrows with annotations highlight pixels from which traces are shown in (**C**,**D**), with the arrow labels corresponding to the row and column indices within the activation map. (**B**) Analogous activation map from a wave pass in the same heart after isoproterenol administration (20 nM). (**C**) Traces of the three pixels highlighted via the red arrows. The black lines show activation times (points of half-maximum activation) as determined by COSMAS. The time shown on the x axis is taken relative to the preceding clock, and the traces were mean-subtracted on the y axis to facilitate their visualisation in a single plot. (**D**) Traces of the same three pixels in the map extracted from the heart after isoproterenol administration.

**Figure 5:**
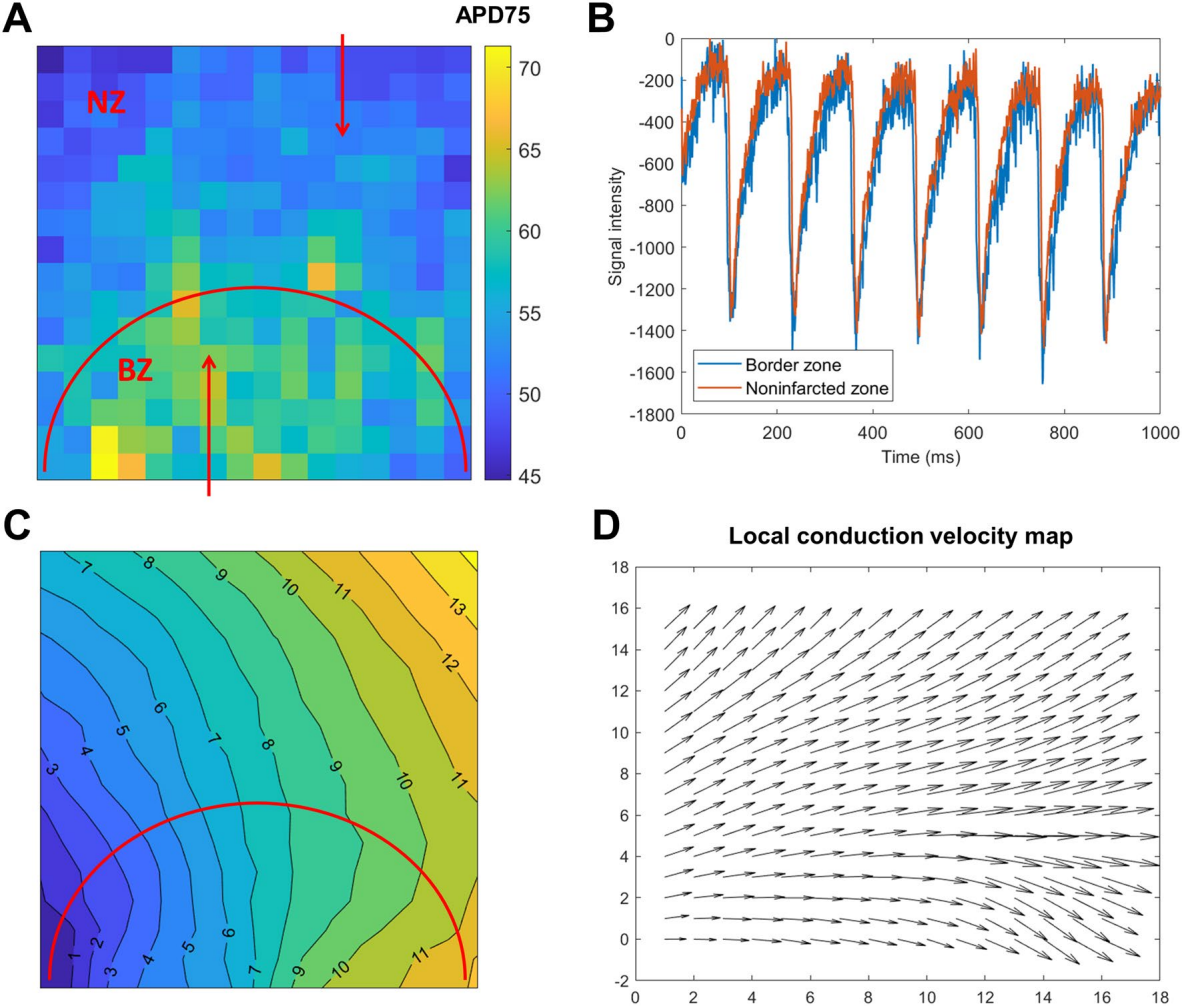
Spatial heterogeneity in rat hearts with a healed infarct. (**A**) A map of AP duration at 80% recovery. The zone under the red semi-circle corresponds to the infarct border zone (BZ), with the zone above the semi-circle being non-infarcted zone. Red arrows point to pixels from which sample traces in the (**B**) panel are extracted. (**B**) Sample traces from border and non-infarcted zone verifying the prolongation of the APs in the border zone (manifesting both earlier depolarization and later depolarization). The traces were scaled to have a similar amplitude and baseline, facilitating visual comparison. (**C**) Average activation map in the same recording. (**D**) Local conduction velocity visualization for panel (**C**), estimated by Bayly’s method^17^, implemented in COSMAS.

**Figure 6:**
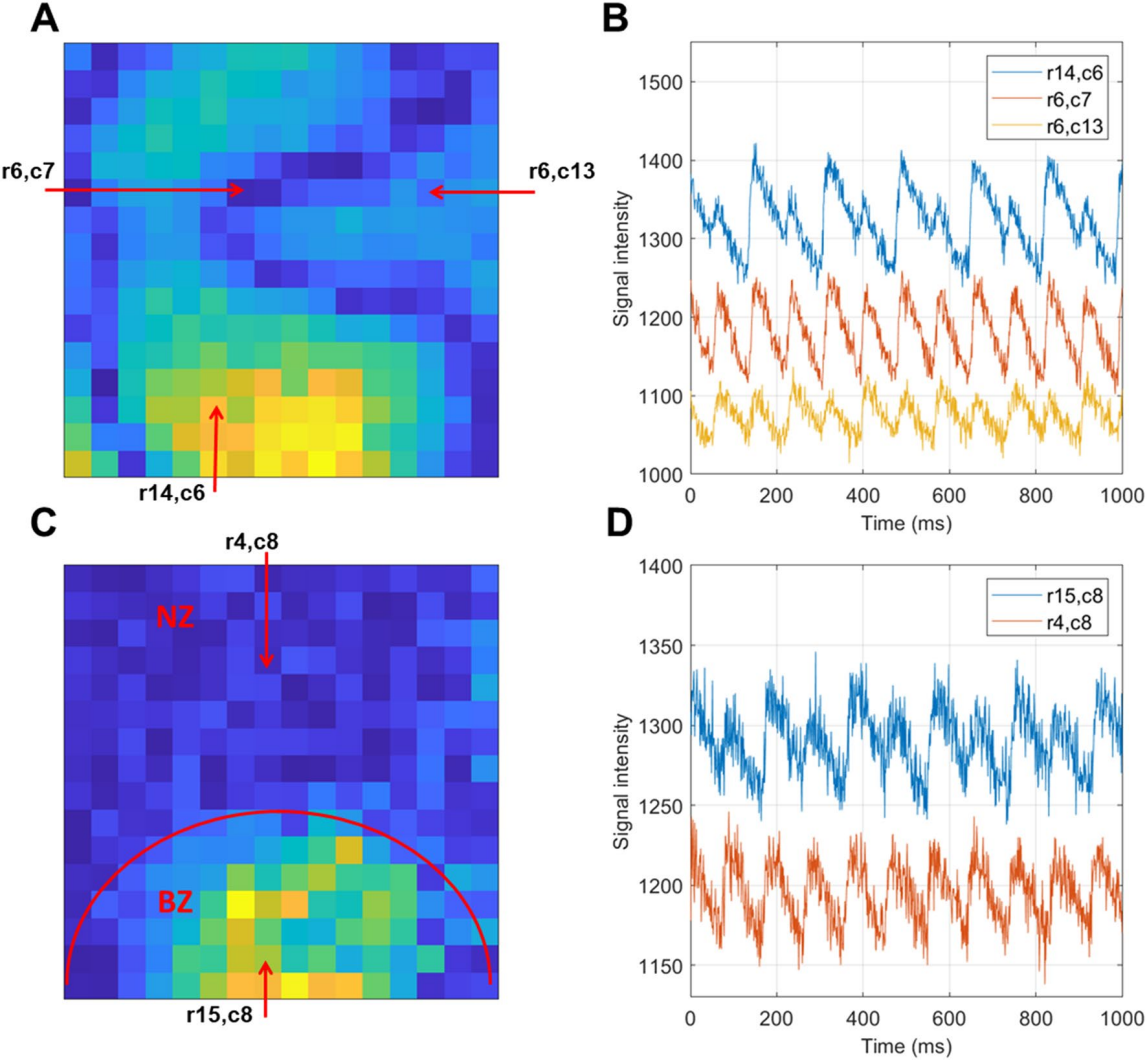
Examples of alternans maps and traces. (**A**) Calcium transient amplitude alternans map corresponding to discordant alternans, with warmer colors corresponding to alternans with large amplitude. Dark blue zones are nodal lines separating zones of alternans in opposite phases. The pixels marked by red arrows with annotations giving the row and column indices are used in the subsequent panel. (**B**) Sample traces from the three marked pixels in the recording in (**A**), verifying that the alternans is discordant (blue and yellow traces have opposite phases) and that there is a lack of alternans in the nodal lines (red trace). (**C**,**D**) Similar format to (**A**,**B**), but showing spatially heterogeneous alternans in an infarcted heart, where alternans arises only in the infarct border zone (BZ) but not the non-infarcted zone (NZ).

**Figure 7:**
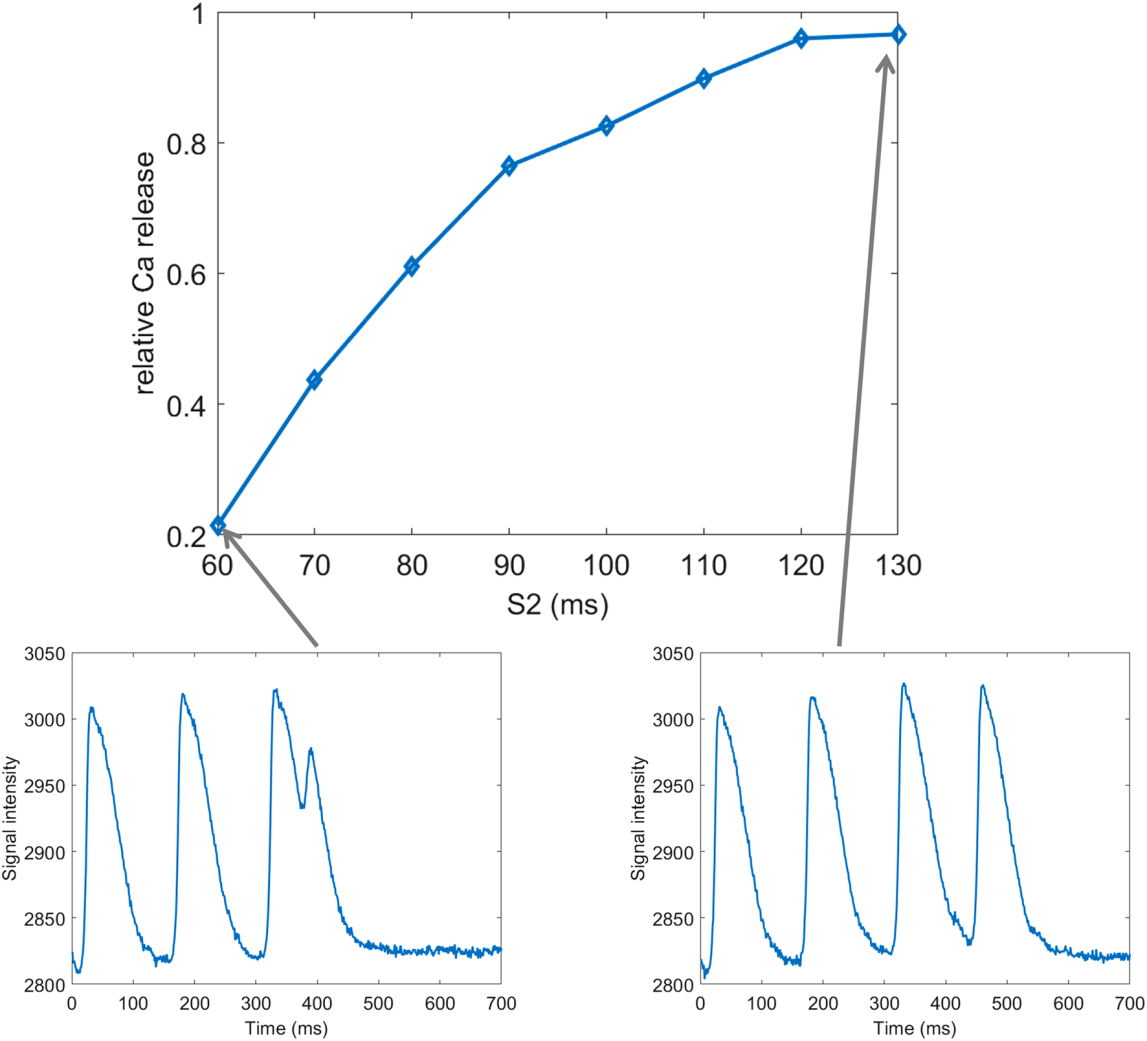
An example of CaT amplitude restitution in S1-S2 protocol. In the top image is shown the relative CaT amplitude of the S2 stimulus versus S1 stimuli, demonstrating that for short S2 coupling interval, the CaT amplitude is reduced. CaT amplitude is defined as the upstroke amplitude (i.e. from the start of the CaT to the peak). Below the restitution curve are two examples of traces from which the curve was extracted: for S2 = 60 ms and S2 = 130 ms. The S1 coupling interval was 150 ms.

Voltage and calcium dye imaging were performed on Langendorff perfused SD rat hearts (300–350 g animal weight) at physiological temperature. The Langendorff heart mapping data in Figs. 2A, 4, 5, 6, 7 are a subset of data used in^9^, with the cited article containing details on experimental and imaging conditions. The hearts were perfused with oxygenated Tyrode solution (flow rate of 10 ml/min; in mM: NaCl 120.3, KCl 4, MgSO_4_ ∗ 7H_2_0 1.3, NaH20P4 1.2, CaCl_2_ 1.2, NaHCO_3_ 25.2, glucose 11). Blebbistatin at the concentration of 10 μM was used to prevent motion artifacts during imaging and pluronic (10% solution, 100 μl per 200 ml of Tyrode solution) was used to aid dye loading. After mechanical uncoupling, a thin plastic tube was inserted to the left ventricle via the left atrial appendage and mitral valve to prevent perfusate pressure build-up. Dyes were injected through a gel membrane into the plastic tube above the perfusion cannula. 100 μl of Rhod-2 (1 mg in 1 ml solution; delivered over 2 min) and 20 μl of RH-237 (5 mg in 1 ml solution; delivered over 1 min) were used . Data in Figs. 1, 3, 9, 10, and Supplementary Figure 2–4 were extracted using the same imaging setup and experimental conditions, except that no calcium dye was used.

Data used to demonstrate noise-resistance of COSMAS in Fig. 2A,B and Supplementary Figure 1 were generated by pacing the ToR-ORd model of human ventricular myocyte^16^ at 2 Hz for 1000 beats, copying the last recorded calcium transient a given number of times to obtain a sequence of calcium transients. The data were subsequently normalized to 0–1 range and additive zero-mean Gaussian noise of the given standard deviation was added.

**Figure 8:**
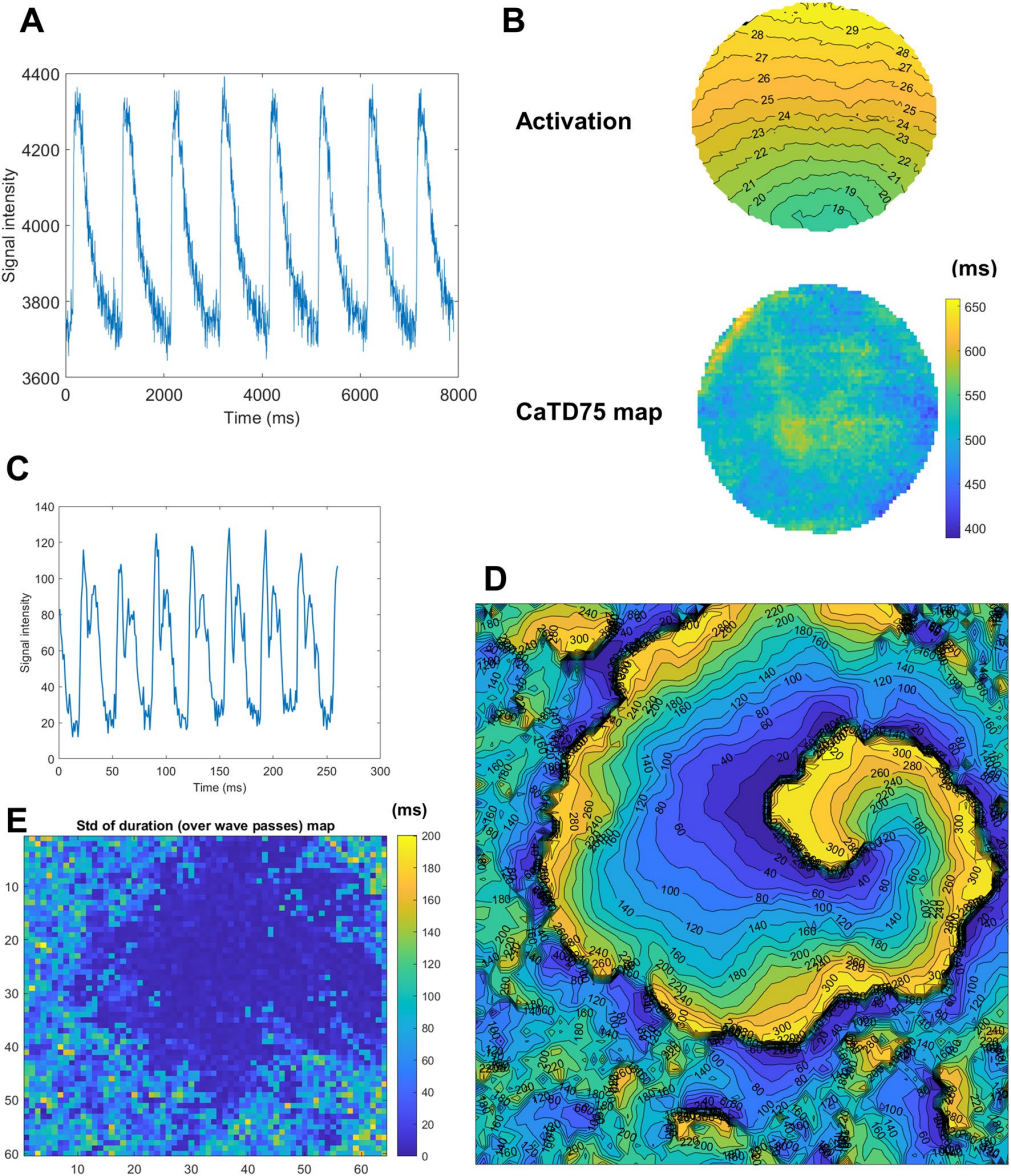
Application of COSMAS to a dish of calcium-imaged monolayer. (**A**) Sample pixel trace (1 Hz pacing). (**B**) Activation map extracted from a culture dish showing calcium-based propagation (top) and a map of CaT duration (CaTD) measured at 80% recovery (bottom). (**C**) Sample trace from a recording obtained off-axis cell culture imaging^19^ showing characteristic “double-hump” motion transient morphology corresponding to cellular contraction and relaxation. (**D**) The activation map of a slow-conducting spiral wave imaged using off-axis imaging, with chaotic culture activation around the spiral. (**E**) The map of temporal standard deviation of duration of cellular activation, computed over multiple wave passes.

The cardiac monolayer data in Fig. 8 were obtained using the protocols described in^18^. In brief, optical mapping was performed on Rhod-4-AM (10 µM, AAT Bioquest, Sunnyvale, CA) treated cardiac monolayers (P1-P3 SD neonatal ventricular rat myocytes). Rhod-4-AM was diluted in tyrodes solution (in mM: NaCl, 135; MgCl_2_ 1; KCl, 5.4; CaCl_2_, 1.5; NaH_2_PO_4_, 0.33; glucose, 5; and HEPES 5 adjusted to pH 7.4 with NaOH). Culture imaging was conducted at room temperature. The spiral wave in Fig. 8 was extracted using the same culturing protocol and solution, but was imaged by measuring contraction artifacts using off-axis illumination as described by Burton et al.^19^.

## Results

Here we present a range of applications of COSMAS to optical mapping data.

### Activation mapping in Langendorff rat hearts

COSMAS may be used to assess the effect of a given treatment on the pattern of activation and conduction velocity. For example, conduction acceleration may be observed after isoproterenol administration in rat hearts (Fig. 4A,B). In Fig. 4C,D are shown three traces before and after isoproterenol administration from pixels highlighted in Fig. 4A, with the activation times detected by COSMAS shown as black lines. Importantly, these are raw traces before any processing (carried out by COSMAS internally to find the activation times shown), showing that the extraction of activation times does not introduce biases/shifts in timing. They also demonstrate that COSMAS can work with noisy signals of varying amplitude.

### Observing heterogeneity in infarcted Langendorff rat hearts

The fact that COSMAS returns maps of various features makes it readily applicable to studies on spatial heterogeneity. For example, a map of AP duration at 75% recovery (APD75) shows that the border zone of a healed infarct in a rat has longer APD75 than the surrounding myocardium (Fig. 5A,B). Activation map extracted from the same recording also shows slower stimulus propagation in the infarct border zone (Fig. 5C,D), which leads to the wavefront curving around the infarct. Reduced conduction velocity in a border zone of a healed infarct is a known hallmark of border zone remodeling associated with downregulation of fast sodium current and myocyte uncoupling^20^.

### Alternans mapping

COSMAS can be used to analyze alternans in cardiac recordings, such as CaT amplitude alternans (Fig. 6). For example, it can reveal nodal lines in the setting of discordant alternans, where the tissue zones on either side of the nodal line oscillate in the opposite phase, with one zone showing a diminished CaT and another an enlarged one^21^ **(**Fig. 6A,B). It can also visualize heterogeneity in alternans magnitude, such as in rat hearts with a healed myocardial infarction, where the border zone is particularly prone to alternans formation^9^ (Fig. 6C,D).

### Custom comb design enables S1S2 protocol analysis

While the simplest form of the comb algorithm serves to process regularly activated recordings (searching for a set of minima in a recording that are a constant number of frames apart), COSMAS also enables the user to provide a custom ‘comb’, where the signal minima to-be-found have intervals of any length between them. This provides the possibility to process recordings with any a priori known activation pattern. For example, for S1-S2 protocol with three S1 = 150 ms stimuli and a single S2 = 60 ms stimulus (Fig. 7, bottom left), the user can construct a comb searching for 3 minima that are 150 ms apart, and then another minimum that is to be found 60 ms after the last S1. Using a custom comb for each distinct S2 coupling interval, features such as signal amplitude or duration may be extracted from S1S2 recordings; an example of S1S2 restitution of CaT amplitude is given in Fig. 7, top. We note that in case of measurements of tissue-level response to S1-S2 protocol, the diastolic intervals may differ somewhat in different regions due to conduction velocity restitution properties. In such a case, the width of local search around the initial positioning of the comb teeth is best increased (via the ‘refinementWidth’ parameter) so that the minima search covers the range of activation times observed in the sample.

### Optical mapping of cell cultures and spiral waves

The applications of COSMAS are not limited to macroscopic Langendorff heart imaging, as presented in previous sections, but can just as well be used to process recordings from calcium-imaged cardiac monolayers without additional customization. Figure 8 illustrates a sample calcium trace in such a culture (Fig. 8A), as well as an activation and an 80% recovery duration map extracted from a dish (Fig. 8B).

While COSMAS is aimed primarily at the analysis of non-arrhythmic phenomena and features, it can also process periodic arrhythmia, such as a reentrant spiral wave (Fig. 8C,D), imaged using the off-axis illumination technique^19^. The spiral wave shown in Fig. 8D was surrounded by highly chaotic wavelets and seemingly random spurious activity, producing fragmented and difficult to interpret activation pattern around the edges of the image. COSMAS is intrinsically capable of detecting such sites of irregular activation in the recording processed (and possibly excluding them from further analysis). This is demonstrated in Fig. 8E, which shows the temporal standard deviation of the estimated APD75, computed over all the spiral wave passes. In sites where the activation is regular, the signal shape is consistent across wave passes, yielding low standard deviation value. Conversely in sites of nonperiodic activation, the comb algorithm does not segment the tissue activation correctly, instead forcing the nonperiodic signal to fit within the periodic comb, producing poor segmentation and thus high temporal standard deviation of the APD75 (or other features) extracted.

### Comparison of key functionality to ElectroMap

**Figure 9:**
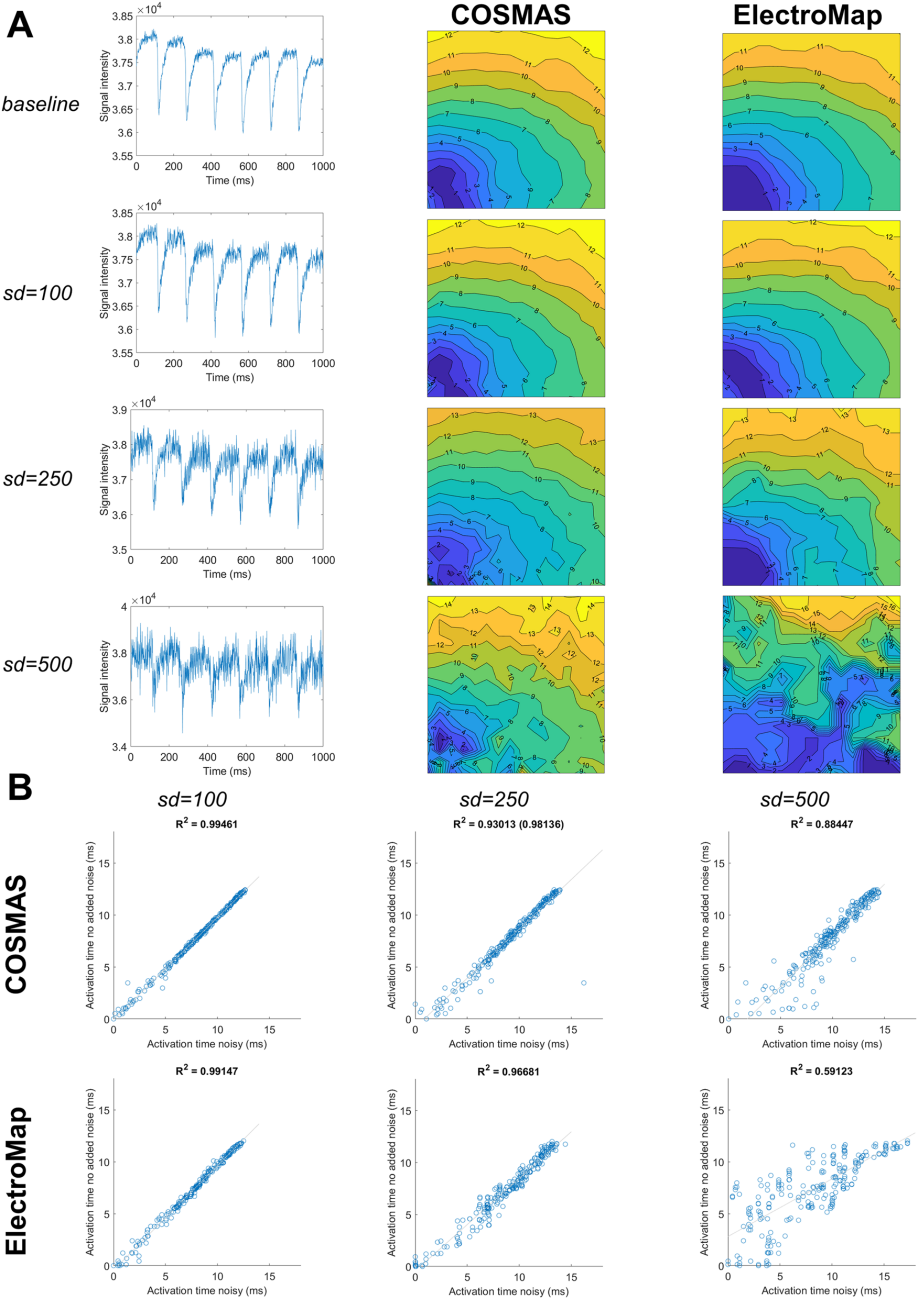
Robustness of COSMAS’ and ElectroMap’s activation mapping to noise. (**A**) The four rows of images correspond to a baseline recording, and then the same recording with three levels of added noise (Gaussian noise with standard deviation of 100, 250, and 500). The first column shows a sample pixel trace from the center of the field of view, the second column gives the activation maps produced by COSMAS, with the third column displaying activation maps produced by ElectroMap. (**B**) A measure of how the extracted activation pattern changes with added noise. In the three columns is shown, for both tools, the correlation of activation times in the noisy recordings (x-axis) versus the activation times in control recording (y-axis). Tight correlation means that there is little change in activation detection with the added noise. Above the correlation plots is given the R^2^ statistic, corresponding to goodness-of-fit. In COSMAS, noise sd = 250, the R^2^ value in parentheses gives the statistic value after removal of one outlier point which perturbs the statistic markedly. Parameters of COSMAS and ElectroMap were matched whenever possible (see Methods for details). The original data with no added noise come from Langendorff-perfused rat hearts paced at 150 ms basic cycle length.

In this section, we compare the COSMAS’ functionality for activation mapping and calcium alternans mapping with that of ElectroMap, a recently published comprehensive software for analysis of optical mapping data^12^. First, we evaluated the robustness of activation mapping to three levels of additional Gaussian noise (Fig. 9A), observing the correlation of recorded activation times after noise addition to the activation times recorded in the baseline recording (Fig. 9B). At baseline conditions with no noise, both tools are in excellent agreement with regards to the activation times (Fig. 9A, R^2^ > 0.99, Supplementary Figure 3). With the addition of light Gaussian noise, standard deviation sd = 100 signal intensity units, the activation pattern was essentially unchanged in both tools, with COSMAS showing marginally tighter correlation to the noise-free activation times (R^2^ = 0.995 vs 0.991 in ElectroMap). With intermediate level noise (sd = 250), the resulting maps are less smooth in both tools (Fig. 9A), and the correlation plots show more frequent deviations from the noise-free activation times (Fig. 9B). COSMAS manifests a slightly worse R^2^ correlation value than ElectroMap in this scenario (R^2^ = 0.93 vs 0.967), but this is driven by a single outlier point (coming from the bottom left corner of the recording which has the poorest signal quality) to which the R^2^ statistic is notoriously sensitive. When the single outlier is removed, COSMAS’ R^2^ is slightly better than that of ElectroMap (0.981 vs 0.967), suggesting that the average deviation from the original recording is lower (which can be also seen in Fig. 9b, 2nd column, where the spread of COSMAS’ points excluding the outlier is smaller than in ElectroMap). With a high level of noise added (sd = 500), while both tools show a markedly deteriorated quality of activation map detection, the COSMAS’ output shows a better resemblance with the original mapping pattern (Fig. 9A). The R^2^ statistic is also markedly better in COSMAS compared to ElectroMap (0.884 vs 0.591, Fig. 9B).

**Figure 10:**
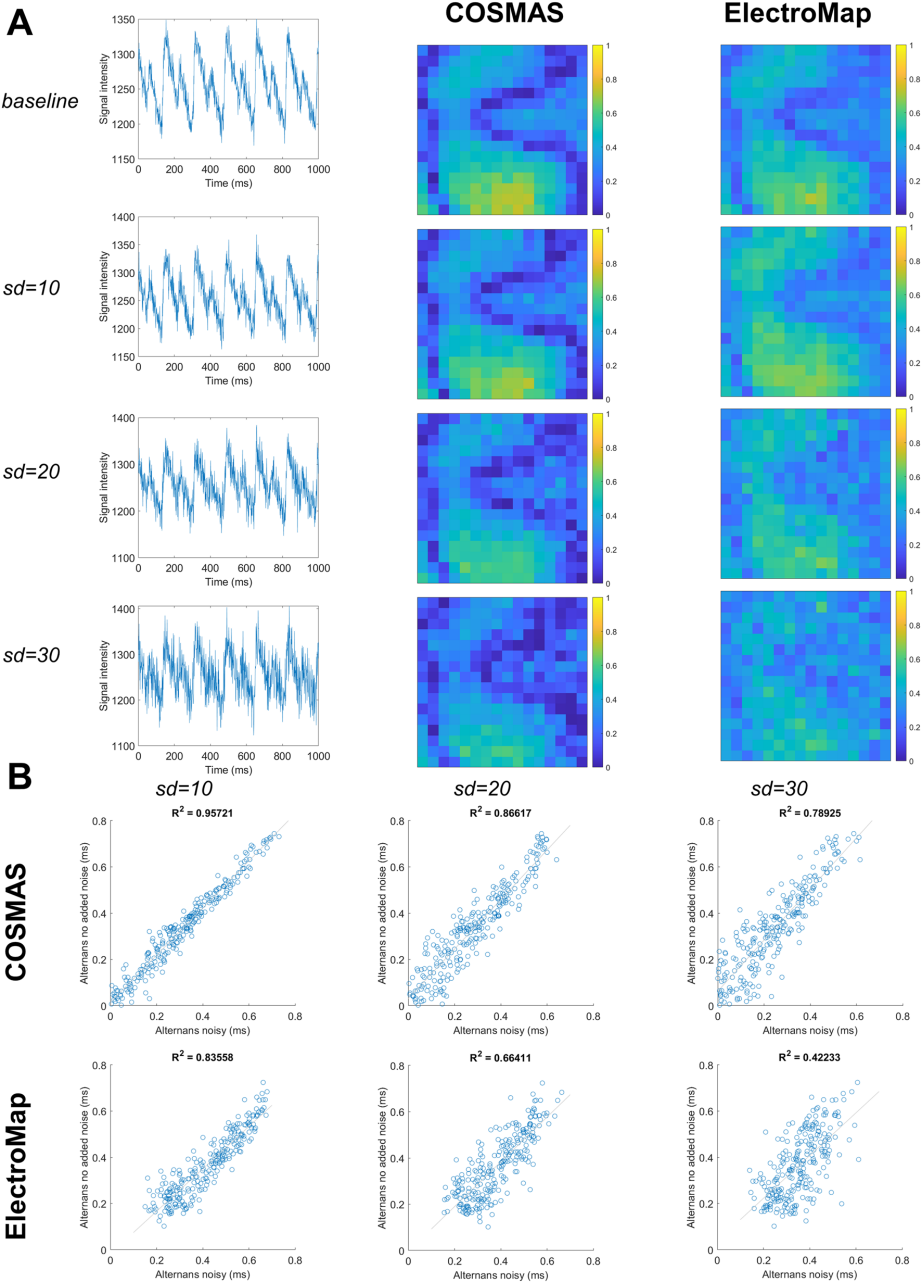
Robustness of COSMAS’ and ElectroMap’s alternans mapping to noise. The structure of the figure is identical to Figure 9. Sample traces in (**A**) come from pixel row = 13, column = 7 in the recording. Given different amplitude of the calcium signal, lower amount of Gaussian noise was applied. Parameters of COSMAS and ElectroMap were matched whenever possible, including the alternans metric (see “Methods” section for details). The original data with no added noise come from Langendorff-perfused rat hearts paced at 85 ms basic cycle length.

Subsequently, we similarly evaluated the robustness of alternans mapping to added Gaussian noise in matching conditions (Fig. 10A). In the recording with no added noise, both tools produce relatively similar outputs, showing the pattern of nodal lines (Fig. 10A, R^2^ = 0.88; Supplementary Figure 4A). However, ElectroMap reports a small degree of alternans even when alternans is not present (Supplementary Figure 4B), leading to a somewhat lower contrast in its alternans maps. COSMAS’ alternans mapping appears relatively resistant to increasing levels of noise, maintaining a stable visual pattern (Fig. 10A, B, R^2^ = 0.957, 0.866, 0.789 for the three levels of added noise versus original recording). While ElectroMap was resistant to a small amount of noise added, the alternans maps deteriorated when noise with standard deviation of 20 or 30 was added (Fig. 10A, B, R^2^ = 0.836, 0.664, 0.422 for the three levels of added noise).

## Discussion

This work presents and demonstrates COSMAS, a tool for automated analysis of optical mapping data from cardiac preparations, such as explanted hearts or cardiac cell cultures grown in monolayers. Two key aims of COSMAS are to provide high-quality processing and to maintain simplicity of methods and code. Unlike previous tools for optical mapping data analysis that were implemented in MATLAB^11,12^, COSMAS is provided both as a MATLAB library and also as a Python library, thus being more accessible to researchers without MATLAB access.

COSMAS offers powerful functionality to extract activation maps, signal duration, amplitude, and baseline, as well as analyze alternans in these features. The quality of processing follows from the use of the comb algorithm^15^, which leverages knowledge of the pattern of activation in the cardiac preparation to reduce the impact of noise, while also avoiding a range of segmentation artefacts that arise in common algorithms of segmentation, such as thresholding. Our comparison of COSMAS and a recently published high-quality software for optical mapping analysis ElectroMap^12^ suggests that with regards to activation mapping, and CaT amplitude mapping, COSMAS performs as well as or better than ElectroMap in common tasks, and is more resistant to noise in the data (Figs. 8, 9). On the other hand, ElectroMap is a more comprehensive tool that allows analysis of several features that are currently not supported in COSMAS (e.g. mapping of time constant of recovery of CaTs) and its GUI-based nature may be helpful for analysis of small datasets.

The relative simplicity of COSMAS is enabled by its focus on the most common tasks in optical mapping, the avoidance of GUI, and the utilization of prior knowledge of the activation rate in the analyzed recordings. The simplicity is advantageous in the following ways:

First, the simplicity arising from script-based design, as opposed to GUI, means that it is easy to write fully automated analyses that can load a whole batch of experiments, analyze them, and then perform statistics or visualization on them to produce publication-ready figures. This not only saves large amounts of time, but it also prevents potential user mistakes such as inconsistent parametrization between recordings and mistakes in storing or loading output, increasing analysis reproducibility. Similar design considerations have driven redesigns in other scientific software platforms^14^.

Second, it means the methodology is transparent. It is tractable why the software produces its outputs and, when compared to more complex methods, potential problems are easier to find and fix. We have a first-hand experience of difficulties arising from complex solutions related to our previous data analysis software Ccoffinn for comprehensive analysis of high-resolution mapping data recorded in cell cultures^22^. In most applications of Ccoffinn by other groups that we are aware of, our help was needed for reading and preprocessing data, for setting optimal parameters, and for interpreting the outputs. We hope that the simplicity of COSMAS makes it a far more approachable tool.

Third, the design and structuring of the codes makes it much easier to extend COSMAS with further functionality compared to software with architecture predetermined by a GUI. Implementing a simple change, such as the addition of a parameter or a pre-processing step, is much more challenging in a GUI-based complex tool. Given that working with MATLAB’s GUI is a relatively specialized skill, we consider a GUI-based design a major limitation with regards to user adaptability of the software.

Ultimately, avoiding complex approaches to signal analysis also means that COSMAS offers excellent computational performance, allowing the processing of hundreds to thousands of recordings per hour on a common PC (depending on hardware and data resolution). The combination of script-based automation and fast runtime makes COSMAS suitable for applications in large-scale drug screening^23,24^.

## Limitations

The most obvious limitation of COSMAS is tightly linked to its reliance on the comb algorithm for segmentation of stimuli: it requires the user to have a prior knowledge of the activation pattern of the recording. At the same time, it can be argued that fixed-rate pacing or other known-pattern protocol (such as S1S2) should be used for most domains of COSMAS’ application, making the limitation relatively theoretical. One obvious example is alternans, which is typically evoked by fixed-rate rapid pacing, but even experiments measuring conduction velocity do benefit from fixed-rate activation. For example, when a drug is suspected to change conduction velocity, but it also changes heart rate, using fixed-rate pacing makes it possible to tease apart the effect of the drug on conduction properties from the effect of conduction velocity restitution.

Ultimately, even for recordings where the pattern of excitation is not controlled by the user (e.g. spontaneously beating cultures in multi-well plates used in high-throughput studies), it may be often very easy to automatically extract the times of activation. This may be achieved, e.g. using thresholding on the spatial average of the whole recording (which tends to be very low-noise in general, allowing simple thresholding to work adequately), and taking the signal maximum in each segment above the threshold as an activation time. A custom comb may be then automatically constructed based on intervals between thus detected activation times (substituting the need of prior knowledge of activation rate). This may then be provided to COSMAS, allowing high-quality segmentation of APs and CaTs for each pixel’s trace in such recordings (for which thresholding would not have worked adequately due to much lower signal-to-noise ratio).

## Supplementary Information

Supplementary Information

## Data Availability

Imaging datasets analysed in the study are available at https://github.com/jtmff/cosmas.

## Code availability

The codes, data, sample scripts illustrating common use cases, and a user guide are available at https://github.com/jtmff/cosmas.
